# Carbonyl Emissions in E-cigarette Aerosol: A Systematic Review and Methodological Considerations

**DOI:** 10.3389/fphys.2017.01119

**Published:** 2018-01-11

**Authors:** Konstantinos E. Farsalinos, Gene Gillman

**Affiliations:** ^1^Department of Cardiology, Onassis Cardiac Surgery Center, Kallithea, Greece; ^2^Department of Pharmacy, University of Patras, Patras, Greece; ^3^National School of Public Health, Athens, Greece; ^4^Enthalpy Analytical, Inc., Durham, NC, United States

**Keywords:** smoking, e-cigarettes, carbonyls, emissions, aerosol

## Abstract

Carbonyl emissions from tobacco cigarettes represent a substantial health risk contributing to smoking-related morbidity and mortality. As expected, this is an important research topic for tobacco harm reduction products, in an attempt to compare the relative risk of these products compared to tobacco cigarettes. In this study, a systematic review of the literature available on PubMed was performed analyzing the studies evaluating carbonyl emissions from e-cigarettes. A total of 32 studies were identified and presented. We identified a large diversity of methodologies, with substantial discrepancies in puffing patterns, aerosol collection and analytical methods as well as reported units of measurements. Such discrepancies make comparisons difficult, and in some cases the accuracy of the findings cannot be determined. Importantly, control for the generation of dry puffs was not performed in the vast majority of studies, particularly in studies using variable power devices, which could result in testing conditions and reported carbonyl levels that have no clinical relevance or context. Some studies have been replicated, verifying the presence of dry puff conditions. Whenever realistic use conditions were ensured, carbonyl emissions from e-cigarettes were substantially lower than tobacco cigarette smoke, while newer generation (bottom-coil, cotton wick) atomizers appeared to emit minimal levels of carbonyls with questionable clinical significance in terms of health risk. However, extremely high levels of carbonyl emissions were reported in some studies, and all these studies need to be replicated because of potentially important health implications.

## Introduction

Tobacco cigarette smoking has well-documented adverse health effects. Due to difficulty in quitting smoking, harm reduction products have been developed in an attempt to help smokers switch to less harmful forms of nicotine intake. Historically, snus has been used as a tobacco harm reduction product; substitution of snus for cigarette smoking has significantly contributed to reducing smoking-related mortality in Sweden (Ramström and Wikmans, 2014). One of the main determinants of the public health effects of a tobacco harm reduction product is its safety/risk profile and levels of toxin exposure, with snus having a documented substantially lower risk compared to smoking (Lee and Hamling, 2009; Vidyasagaran et al., 2016).

E-cigarettes were invented in recent years, but awareness and use has grown exponentially. They are currently considered the most popular tobacco harm reduction product among smokers. Limited research exists on the epidemiological effects of e-cigarettes; thus most research is focused on chemical and toxicological assessment (Farsalinos and Polosa, 2014). Carbonyl emissions from e-cigarettes represent a research subject that has generated a lot of interest. High levels of carbonyls are emitted in tobacco cigarette smoke, mainly derived from the thermal degradation of sugars due to the high temperatures of combustion during smoking (Rustemeier et al., 2002; Counts et al., 2005; Baker et al., 2006; Paschke et al., 2014). Formaldehyde is classified as a group 1 carcinogen for humans by the International Agency for Research on Cancer while other carbonyls such as acrolein and acetaldehyde are also listed as toxic or carcinogenic (US OSHA, 2007, 2011). The main ingredients in e-cigarette liquids, propylene glycol (PG) and glycerol (VG) are known to be oxidized to carbonyls (Bekki et al., 2014; Spencer and Lauterbach, 2015). As a result, evaluating carbonyl emissions from e-cigarettes is an important step in determining the both the absolute and relative (to smoking) risk of e-cigarettes, especially considering the variability of performance characteristics designs and functional patterns of different e-cigarette devices. The purpose of this study was to perform a systematic review of the literature on carbonyl emissions from e-cigarettes.

## Methods

**Figure 1:**
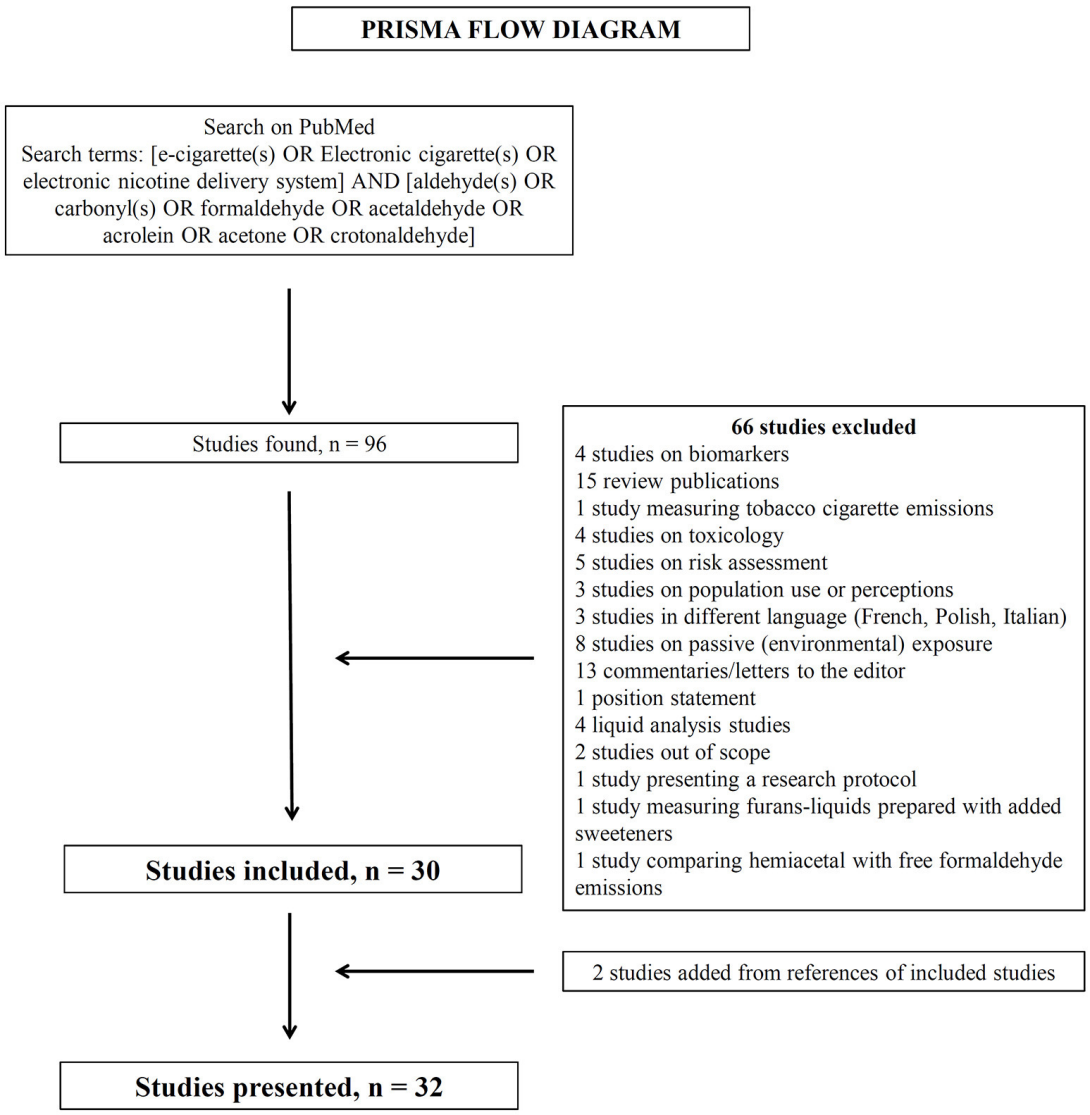
Prisma flow diagram showing the methodology for literature review and selection of studies.

This systematic review was performed through a search on PubMed electronic database for English language articles without any date restriction. This review focused on the main toxic carbonyls that are found at high levels in tobacco cigarette smoke, namely formaldehyde, acetaldehyde, acetone, acrolein, and crotonaldehyde. The search terms on PubMed (title and/or abstract) were: [e-cigarette(s) OR Electronic cigarette(s) OR electronic nicotine delivery system] AND [aldehyde(s) OR carbonyl(s) OR formaldehyde OR acetaldehyde OR acrolein OR acetone OR crotonaldehyde]. The Prisma Flow Diagram for the search is shown in Figure 1. The PubMed search resulted in 96 studies. After careful review of the titles, abstracts and full text, 66 studies were excluded, while two additional studies (which did not include the terms of the search in the title or abstract) was found from the citations of other studies. The current review presents the findings from 32 published studies.

### Published studies on carbonyl emissions from e-cigarettes

Uchiyama et al. (2010) analyzed an e-cigarette from the Japanese market for the presence of carbonyls in the aerosol. They used coupled silica cartridges impregnated with hydroquinone and 2,3 dinitrophenylhydrazine (DNPH) to trap carbonyls, and analysis was performed with high-performance liquid chromatography (HPLC). The levels of carbonyl emissions were reported as amount per m^3^. A puff flow rate of 500 mL/min was reported, but no information on puff duration and interpuff interval was provided. Formaldehyde was detected at levels of 8.3 mg/m^3^, acetaldehyde at 11 mg/m^3^, acetone at 2.9 mg/m^3^, and acrolein at 9.3 mg/m^3^.

Uchiyama et al. (2013) analyzed 13 brands of e-cigarettes for the levels of carbonyl emissions using coupled silica cartridges impregnated with hydroquinone and DNPH. The analysis was performed with HPLC. The e-cigarettes were puffed based on Health Canada Intense puffing regime (55 mL puff volume, 2 s puff duration, 30 s interpuff interval) and the levels were reported as amount per m^3^. Formaldehyde levels varied from non-detected to 61 mg/m3, acetaldehyde from non-detected to 48 mg/ m^3^, and acrolein from non-detected to 36 mg/ m^3^. Other carbonyls such as propanal, and glyoxal were also detected in some products. The authors noted that large variations in carbonyl levels were detected, not only among different brands but also among different samples of the same brand, while 4 of the 13 brands did not generate any carbonyl emissions above the method detection limit.

Goniewicz et al. (2014) tested 12 different e-cigarette brands, in most cases first generation products that are today considered outdated. They also tested a medicinal nicotine inhalator as reference product. A relatively short puff duration (1.8 s) and interpuff interval (10 s) was used, while the puff volume was 70 mL. Carbonyls were trapped in tubes packed with solid adsorbent and analysis was performed by HPLC with diode array detector (HPLC-DAD). The study detected 4 of the 15 carbonyls that were tested. Values, expressed in amount per 150 puffs, ranged from 3.2 to 56.1 μg for formaldehyde (0.021–0.374 μg/puff), 2.0 to 12.0 μg for acetaldehyde (0.013–0.080 μg/puff), non-detected to 41.9 μg for acrolein (0.279 μg/puff) and 1.7 to 7.1 μg for o-methylbenzaldehyde (0.011–0.047 μg/puff). Small amounts of formaldehyde, acetaldehyde and o-methylbenzaldehyde were also found in the nicotine inhalator. The authors compared the findings with literature data and on tobacco cigarettes and reported that carbonyl emissions were 9- to 450-fold lower in e-cigarettes.

The same research group performed a second study measuring carbonyl emissions from 10 commercially available liquids using different voltage settings (3.2, 4.0, and 4.8 V) in a variable-voltage e-cigarette battery device (Kosmider et al., 2014). Also, different mixtures of e-cigarette liquid solvents (PG, VG and a mixture of both) without flavoring, proprietary prepared by the researchers, were tested. The authors used a now-outdated CE4-type (top coil, silica wick) atomizer. Aerosol was generated at 1.8 s puff duration and 17 s interpuff interval, while puff volume was 70 mL. Carbonyls were trapped in tubes packed with solid adsorbent and analysis was performed by HPLC with diode array detector (HPLC-DAD) and levels were reported as amount per 15 puffs. Additionally, the battery button was manually activated 1 s before the puff was taken. At least one carbonyl compound was detected in all samples. Formaldehyde levels ranged from non-detected to 59 ng/15 puffs (3.99 ng/puff), acetaldehyde from non-detected to 107 ng/15 puffs (7.11 ng/puff) and acetone from non-detected to 296 ng/15 puffs (19.73 ng/puff). Acrolein and crotonaldehyde were not detected in any sample, while other carbonyls such as butanal, isovaleric aldehyde, and m-methylbenzaldehyde were detected in some samples. The authors identified that higher levels of carbonyls were emitted from PG compared to VG-based liquids. Additionally, carbonyl emissions increased at 4.8 V by 4- to 200-fold compared to emissions at 3.2 V.

Hutzler et al. (2014) tested 7 commercial liquids for the presence of carbonyls. Initially, the authors incubated the liquids in headspace gas chromatography-mass spectrometry (GC-MS) at various temperatures for 2 h. They reported an increase in formaldehyde (up to 10- to 20-fold) and acetaldehyde levels (up to 700-fold) at 150°C incubation temperature compared to 100°C. Subsequently, they used a smoking machine and generated aerosol using a first generation (“cigalike”) e-cigarette device using a puffing regime of 55 mL puff volume, 3 s puff duration and 30 s interpuff interval. Aerosol production and collection (in impingers containing DNPH) was performed in discreet 10-puff blocks (after an initial 50-puff block) and continued until no visible aerosol was released from the cartridges. Analysis was performed with HPLC-DAD. The authors identified high levels of carbonyls which reached or exceeded the respective levels in tobacco cigarettes during the later puff blocks, reaching to ~5 μg/puff for formaldehyde, 8 μg/puff for acetaldehyde and 3.5 μg/puff for acrolein. This was attributed to the lower liquid levels within the cartridges.

Tayyarah and Long (2014) compared carbonyl emissions from 5 e-cigarette (“cigalike”) products (2 disposable and 3 rechargeable) with 3 tobacco cigarette products. Health Canada Intense puffing regime was used (55 mL volume, 2 s duration and 30 s interval). Aerosol was collected in two impingers connected in series containing DNPH, and analysis was performed with Ultra Performance Liquid Chromatography with ultraviolet detection (UPLC-UV). Formaldehyde was not detected in any of the products, while acetaldehyde was detected at levels of 0.32 μg/puff in 1 product and acrolein was detected in 2 products at levels up to 0.19 μg/puff. Propionaldehyde was also detected in 1 product at levels of 0.11 μg/puff. The levels found were reported to be 86- to 544-fold lower than tobacco cigarette smoke.

Geiss et al. (2015) tested carbonyl emissions from 2 commercial e-cigarettes. The puffing regime was 35 mL volume, 4 s duration and 30 s interpuff interval. They used a 2 L Tedlar gas-sampling bag to collect aerosol generated through a smoking machine and then the aerosol was passed through DNPH-silica cartridges. Analysis was performed using HPLC-DAD. Levels ranged from 19.6 to 23.5 ng/puff for formaldehyde, 8.1 to 39.9 ng/puff for acetaldehyde, 2.7 to 8.8 ng/puff for acetone and 0.5 to 13.5 ng/puff for acrolein. Contrary to Kosmider et al. (2014), higher levels of carbonyls were observed in the VG-based liquid compared to a mixed PG-VG liquid.

In a study that generated a lot of publicity, Jensen et al. (2015) tested a “tank system” e-cigarette with a commercial e-cigarette liquid (Halo “café mocha” flavor, 6 mg/mL nicotine concentration) for the presence of formaldehyde hemiacetals. Hemiacetals are compounds formed from the reaction of PG or VG with formaldehyde. The authors tested two voltage settings (3.3 V and 5.0 V) and used NMR spectroscopy to measure the compounds. The puffing regime was 50 mL volume, 4 s duration and 30 s interpuff interval. No formaldehyde hemiacetals were detected at 3.3 V, while at 5.0 V a mean level of 380 μg/10 puffs was detected. Despite mentioning that the behavior of formaldehyde hemiacetals in the respiratory tract are unknown, they assumed that the risk is similar to formaldehyde and reported that the cancer risk of long term vaping was “5 times as high… or even 15 times as high… as the risk associated with long term smoking” when comparing 3 mL liquid consumption with 20 tobacco cigarettes.

Laugesen (2015) tested 14 e-cigarette products purchased online from China, USA, and UK. Twelve of the products were first-generation (“cigalikes”) while two were tank systems. The puffing protocol was 70 mL puff volume, 3 s puff duration and 10 s interpuff interval. Aerosol was collected in two impingers connected in series that contained DNPH and analysis was performed with HPLC with ultraviolet detection (HPLC-UV). Levels of formaldehyde ranged from 0.48 to 2.5 μg/L of aerosol volume, acetaldehyde from 0.58 to 1.52 μg/L and acrolein from 0.4 to 2.1 μg/L. The authors reported that the levels of carbonyls were 100- to 2,800-fold lower compared to the smoke of a commercial tobacco cigarette.

Farsalinos et al. (2015) measured carbonyl emissions from a new-generation (rebuildable tank) atomizer at different power settings. Two samples of the atomizer were prepared, one with double wick (silica) and the other with single wick. The later was intentionally prepared to generate overheating conditions (dry puffs) at low power settings compared to the other atomizer. For the first time in a study measuring carbonyl emissions in e-cigarette aerosol, experienced vapers were recruited and tested the atomizers to detect and report the power settings associated with dry puffs (discussed below). Power settings from 6.5 to 10 W were tested, and emissions were substantially lower with the double-wick compared to the single-wick atomizer. The puffing protocol was 60 mL puff volume, 4 s puff duration and 30 s interpuff interval. Aerosol was collected in two impingers connected in series that contained DNPH and analysis was performed with HPLC with ultraviolet detection (HPLC-UV). At 10 W, up to 30-fold higher formaldehyde, 50-fold higher acetaldehyde and 200-fold higher acrolein was emitted from the less efficient atomizer, which was identified as generating dry puff at this power setting. Under normal vaping conditions, low carbonyl levels were detected, with formaldehyde up to 11 μg/10 puffs, acetaldehyde up to 4.5 μg/10 puffs and acrolein up to 1 μg/10 puffs. The levels were 7- to 300-fold lower compared to literature data on tobacco cigarette smoke. The authors concluded that, under verified realistic (no dry puff) conditions, e-cigarettes emit low levels of carbonyls.

Herrington and Myers (2015) evaluated 4 commercially available first generation e-cigarettes. They used a manually handled gas-tight syringe to collect aerosol in thermal desorption tubes using 40 mL puff volume, 4 s puff duration and 10 s interpuff interval. The thermal desorption tubes were then transferred to a thermal desorption unit coupled with a GC-MS analytical system. Analysis was performed using Thermal Desorption Gas Chromatography Mass Spectroscopy (TD-GC-MS). The authors verified the presence of several carbonyls in the aerosol such as formaldehyde, acetaldehyde, acrolein, and acetone. However, they did not report the amount of carbonyls emitted with the exception of acrolein which was found at levels of 1.5–6.7 ppm_v_ per 40 mL puff.

Blair et al. (2015) developed a fast-flow tube system that would allow the real time measurements of volatile organic compounds using a proton transfer reaction time-of-flight mass spectrometer (PTRMS). A puff volume of 43 mL, puff duration of 2 s and interpuff interval ranging from 15 to 60 s was used. Aerosol was collected in a Teflon bag and a fast-flow tube setup was prepared. Two e-cigarette products were tested, and most probably they were first-generation products (although that was not clear from the publication). The authors reported acetaldehyde levels at 95.9 μg/9 puffs, acetone at 22.0 μg/9 puffs and acrolein at 32 μg/9 puffs. Several standardized and commercial tobacco cigarettes were also analyze, with acetaldehyde levels being 3- to 6-fold higher, acetone 7- to 15-fold higher and acrolein up to 2-fold higher.

Talih et al. (2016) tested a “dripping” atomizer (a product that does not contain a tank but needs to regularly “drip” liquid from the mouthpiece in order to keep the wick wet). A very old and now-outdated dripping atomizer was used. The authors added 2 drops of e-cigarette liquid and took 2–4 puffs before refilling the atomizer. An extreme 8 s puff volume was used for aerosol generation while puff volume and interpuff interval were set at 152.8 mL and 10 s, respectively. The aerosol was collected in DNPH-coated silica cartridges and carbonyls were analyzed with HPLC-MS. Temperature measurements were also performed, using an infrared camera, and ranged from 130°C (during the first two puffs) to 340°C (at the 4th puff). Interestingly, the temperature was inversely correlated with aerosol yield (liquid consumption per puff), with the 4th puff delivering 3-fold less aerosol compared to the 1st puff and having the highest temperature. Expectedly, temperature correlated with carbonyl emissions. Formaldehyde was detected at levels from 19.7 to 88.06 μg/15 puffs, acetaldehyde at 269.35 to1172.23 μg/15 puffs, acetone at 22.28 to 196.55 μg/15 puffs and acrolein at non-detected to 1.97 μg/15 puffs. The levels reported exceeded in some cases the emissions from tobacco cigarettes.

Flora et al. (2016) examined the aerosol of 4 variants of a commercially available first-generation e-cigarette. The puffing protocol was 55 mL puff volume, 4 s puff duration and 30 s interpuff interval. Aerosol from 20 puffs was collected in two impingers connected in series that contained DNPH and analysis was performed with UPLC-UV. Formaldehyde was detected at levels from 0.09 to 0.33 μg/puff while acetaldehyde was detected below the LOQ (<0.71 μg/puff). Acrolein and crotonaldehyde were not detected in the aerosol.

Gillman et al. (2016) tested 5 refillable tank-type e-cigarette devices at different power settings for carbonyl emissions. Devices included an outdated top coil, silica wick atomizer (“CE4”) which had been used in a previous study (Jensen et al., 2015) and some newer generation bottom coil, cotton wick atomizers. The authors presented in detail the characteristics of each device tested and reported that the minimum level of liquid allowed in the atomizer during the aerosol collection was at 50% of the tank capacity. A proprietary liquid composed of PG, VG, and nicotine (no flavorings) was used in the study. Power settings ranged from 5.2 to 25 W. Four power settings were tested with each atomizer. A smoking machine was used to generate aerosol and the puffing regime was 55 mL puff volume, 4 s puff duration and 30 s interpuff interval. The authors also weighed the atomizer before and after aerosol collection in order to determine liquid consumption, and carbonyl emissions were reported per g of liquid consumption (they also reported levels as amount per puff). A substantial variability in carbonyl emissions was observed between atomizers. Newer generation atomizers emitted formaldehyde from 0.02 to 0.08 mg/g, acetaldehyde from 0.006 to 0.08 mg/g and acrolein from non-detected to 0.06 mg/g. The CE4 atomizer released orders of magnitude higher carbonyl levels compared to other atomizers, with formaldehyde ranging from 2.1 to 7.3 mg/g, acetaldehyde from 1.7 to 5.8 mg/g and acrolein from 0.05 to 0.78 mg/g. Large variability in liquid consumption per puff was observed between different atomizers and power settings, ranging from 1.5 to 28 mg per puff. The authors explained that when higher power resulted in substantially increased liquid consumption per puff, the levels of carbonyls remained low. Contrary to that, smaller increases in liquid consumption per puff were associated increased carbonyl emissions, probably due to liquid overheating and decomposition of PG and VG. Finally, the authors explained that the actual exposure is also limited by the dry puff phenomenon causing an unpleasant taste that users detect and avoid.

Jo and Kim (2016) tested an e-cigarette available in the Korean market for the present of carbonyls in the aerosol. The puff volume was 33.4 mL, the puff duration 2 s and the interpuff interval 10 s. Five, ten, and fifteen puffs per collection were obtained. Carbonyls were trapped in DNPH cartridges, and analyzed using HPLC-UV. In general, low levels of aldehydes were detected (reported as amount per volume of e-liquid), with formaldehyde ranging from 2.03 to 9.17 μg/mL, acetaldehyde from 7.76 to 14.4 μg/mL and acetone from 0.65 to 1.26 μg/mL. Acrolein was not detected in any of the samples. The authors reported that formaldehyde and acetaldehyde were substantially higher in the aerosol compared to the liquid, which is expected since the main source of these compounds is the thermal degradation of PG and VG.

Geiss et al. (2016) tested a new generation, variable power, e-cigarette device at different power settings (from 5 to 25 W) with a commercial liquid to determine carbonyl emissions. Additionally, the temperature of the coil was monitored by infrared thermography and an experienced vaper provided feedback on the subjective quality of the emitted aerosol. The puff volume was 50 mL, the puff duration 3 s and the interpuff interval 20 s. Carbonyls were trapped on cartridges filled with DNPH-coated silica gel adsorbent and analysis was performed by HPLC/UV. Of note, different cartridges were tested and some created significant pressure drop which interferes with the airflow through the e-cigarette device and thus are unsuitable for collecting aerosol from e-cigarettes. The authors found that aldehyde emissions increased steeply from 15 W upwards with a further steep increase at 20 W; however, the vaper identified as borderline the taste at 15 W and perceived the flavor as different and the vapor as too hot from 20 W upwards. At 20 W, the temperature of the coil exceeded 300°C. Formaldehyde levels ranged from 24.2 to 1599.9 ng/puff, acetaldehyde from 13.2 to 348.4 ng/puff and acrolein from non-detected to 2.5 ng/puff (the latter at 25 W only). Tobacco cigarettes emitted 7-fold higher formaldehyde and 600-fold higher acetaldehyde levels compared to the e-cigarette at 15 W.

Uchiyama et al. (2016) evaluated carbonyl emissions from 10 brands of second-generation e-cigarettes available in Japan. Aerosol was generated using Health Canada Intense puffing regime and was collected with a Cambridge filter and sorbent cartridge packed with Carboxen-572 particles connected in series. The puff volume was 55 mL, the puff duration 2 s and the interpuff interval 30 s. Analysis was performed by HPLC-UV. The authors noted that aldehyde emissions increased after the first 11–15 puffs and then reached a steady-state. They also reported substantial increases in carbonyl emissions above 4.0 V, while from 3.2 to 4.0 V carbonyl emissions were very low. Of note, aerosol yield gradually increased at higher voltage setting but decreased from 4.4 to 4.8 V, a clear indication of insufficient liquid in the coil that can generate dry puff conditions (Farsalinos et al., 2015; Gillman et al., 2016). Formaldehyde ranged from non-detected to 790 μg/10 puffs, acetaldehyde from non-detected to 520 μg/10 puffs, acetone from non-detected to 64 μg/10 puffs and acrolein from non-detected to 99 μg/10 puffs. Other carbonyls such as glyoxal and methylglyoxal were also detected.

Havel et al. (2017) measured carbonyl emissions from several e-cigarette products at different voltage settings. An unflavored liquid was used in the experiments and aerosol was generated at 3.0 V (6.0 W), 3.5 V (8.2 W), 4.0 V (10.7 W), 5.0 V (16.7 W), and 5.9 V (23.2 W). The puffing regime was 80 mL puff volume, 4 s puff duration and 30 s interpuff interval. Aerosol was collected in 3 impinger connected in series that contained DNPH and analysis was performed with HPLC-UV. The authors did not report the values of carbonyl emissions but presented a graph (values in μg, probably per collection −15 puffs) showing that carbonyl emissions (formaldehyde, acetaldehyde and acrolein) increased substantially at 5.0 V (16.7 W) and 5.9 V (23.2 W).

Sleiman et al. (2016) two types of e-cigarette devices, a top-coil silica wick atomizer and a bottom-coil silica wick atomizer, with a commercial tobacco-flavored liquid. The puffing regime was 50 mL volume, 5 s duration and 30 s interpuff interval. Carbonyls were trapped in DNPH cartridges (1–5 puffs per collection) and analysis was performed with HPLC-UV. The authors also measured aerosol temperature at the exit of the atomizer and found that the temperature increased after the first 20 puffs. Thus, they tested carbonyl emissions during the first 5 puffs and after the 30th puff (“steady-state” condition). The authors reported findings (in amount per mg liquid consumption) at 3.8 and 4.8 V with the first and at 3.8 V with the second of the atomizer. Remarkably high levels of carbonyls were found at steady-state, with formaldehyde ranging from 1,300 to 48,200 ng/mg, acetaldehyde from 260 to 19,080 ng/mg, acrolein from 120 to 10,060 ng/mg, acetone from 70 to 1,410 ng/mg and crotonaldehyde from 10 to 720 ng/mg. In most cases, the levels exceeded by far the respective emissions from tobacco cigarettes that have been reported in the literature (Counts et al., 2005).

El-Hellani et al. (2016) tested 12 products from 10 brands, including disposable and pre-filled first generation e-cigarettes as well as tank-system atomizers. Different nicotine concentrations and flavoring were chosen, with a total of 29 samples examined. The puffing regime was 100 mL volume, 4 s duration and 10 s interpuff interval. Aerosol passed through silica sorbent tubes coated with DNPH and analysis was performed with HPLC. Total carbonyls ranged from 3.06 to 48.85 μg/15 puffs, with the average levels being 10.52 μg/15 puffs. Formaldehyde levels ranged from 0.87 to 7.57 μg/15 puffs, acetaldehyde from 0.67 to 31.80 μg/15 puffs, acetone from 1.07 to 5.16 μg/15 puffs and acrolein from non-detected to 2.09 μg/15 puffs. The authors reported that carbonyl levels correlated with power settings and were lower compared to tobacco cigarette smoke.

Khlystov and Samburova (2016) examined the difference in carbonyl emissions between flavored and unflavored liquids. Two different e-cigarette atomizers (a top-coil and a bottom-coil, both with silica wick) were tested with various flavored and an unflavored liquid, with the latter containing similar proportion of PG and VG as the former. A third device (a first-generation, cigarette-like battery with prefilled cartomizers) was also tested with flavored liquids only. The puffing regime was 40 mL volume, 4 s duration and 30 s interpuff interval. The authors collected the aerosol of 2 puffs through DNPH cartridges after 15 “warm-up” puffs were obtained (but not collected). Analysis was performed using HPLC. Carbonyls were below the level of detection in unflavored liquids. Carbonyl emissions varied between flavored liquids and in some cases were remarkably high, especially for one of the liquid brands tested (“Brand I”). Formaldehyde ranged from 34.8 to 49.5 μg/puff, acetaldehyde from 18.63 to 27.7 μg/puff and acrolein from 1.31 to 3.44 μg/puff. Based on the liquid consumption per puff reported to the authors, the corresponding values per g liquid consumption were up to 7210 μg/g for formaldehyde, 3631 μg/g for acetaldehyde and 346 μg/g for acrolein.

Wang et al. (2017) examined how carbonyl emissions are affected by the e-cigarette solvent (PG or VG) and the temperature of evaporation. Instead of using an e-cigarette battery device and atomizer, they used a tubular reactor to evaporate two commercial e-cigarette liquids and custom preparations of PG, VG and a mixture of the two (in 1:1 ratio). The liquid (5–10 mg) was impregnated in a glass wool piece and introduced into the reactor. Subsequently, the reactor was introduced into a furnace with temperature set through a controller. The puff flow rate was 200 mL/min, corresponding to a transition time of e-liquid with air in the reactor of 2.9 s (mimicking a 3 s puff). Subsequently, the aerosol passed through 2 DNPH cartridges connected in series. Analysis was performed using HPLC-DAD. The authors found that carbonyl emissions started to increase considerably above 215°C for PG, although the steepest increase was observed above 270°C. The level of formaldehyde was 0.03 μg/mg PG, 0.29 μg/mg PG and 2.03 μg/mg PG at 215°C, 270°C and 318°C respectively. For acetaldehyde the respective levels were 0.03, μg/mg PG, 0.30 μg/mg PG, and 2.35 μg/mg PG. No acrolein was detected when testing the PG liquid. Evaporation of VG liquid resulted in higher levels of carbonyls generated at lower temperatures compared to PG. Additionally, acrolein was detected at 270°C when testing the VG liquid. At 270°C, 27-fold higher formaldehyde and 5-fold higher acetaldehyde was detected with the VG compared to PG liquid. More complex reactions occurred when testing the PG/VG mixture. The test of commercial liquids verified the findings of the PG and VG liquids, with the authors concluding that PG and GL were likely to be the primary sources of emitted carbonyls from these two commercial liquids.

Flora et al. (2017) tested 6 commercially-available first generation e-cigarette devices for carbonyls in the aerosol. The puffing regime was 55 mL volume, 4 s duration, and 30 s interpuff interval. Aerosol passed through a Cambridge filter and then through an impinger containing DNPH. After aerosol collection, the Cambridge filter was inserted into the DNPH trapping solution to derivatize the particulate phase carbonyls. Analysis was performed using UPLC-MS. Substantial variability between different products and between different samples from the same product was detected. Formaldehyde levels ranged from 0.07 to 14.1 μg/puff, acetaldehyde from 0.03 to 13.61 μg/puff, acrolein from below limit of quantification (LOQ) to 4.11 μg/puff and crotonaldehyde from non-detected to 0.04 μg/puff. The authors also assessed the effect of temperature of evaporation on formaldehyde emissions using an infrared camera, and reported that formaldehyde emissions were low at temperatures below 350°C but rose steeply with increasing temperature.

Ogunwale et al. (2017) tested 4 e-cigarette products and 6 liquids using a second generation device composed of a refillable tank-type atomizer (EVOD 2 atomizer) and a variable voltage battery (iTaste VV V3.0). The power of the variable voltage device varied from 9.1 to 16.6 W (3.3–5.0 V). The puffing regime was 91 mL volume, 4 s duration, and 30 s interpuff interval. Aerosol was collected in Tedlar bags and subsequently passed through silicon microreactors with a coating phase of 4-(2-aminooxyethyl)-morpholin-4-ium chloride (AMAH). AMAH–aldehyde adducts were measured using GC-MS while ^1^H nuclear magnetic resonance spectroscopy was used to analyze hemiacetals in the aerosols. Formaldehyde levels ranged from 0.18 to 74.0 μg/10 puffs, acetaldehyde from 0.15 to 63.1 μg/10 puffs, acrolein from 0.02 to 5.8 μg/10 puffs and acetone from 1.29 to 12.5 μg/10 puffs. For the second generation device, the levels were much higher at 16.6 W, reaching to levels of 819.81 μg/10 puffs for formaldehyde, 532.10 μg/10 puffs for acetaldehyde, 16.21 μg/10 puffs for acrolein and 808.72 μg/10 puffs for acetone. Formaldehyde hemiacetals were detected only with one liquid using the second generation device at high power (11.7 and 16.6 W).

Sala et al. (2017) presented a solid-phase microextraction (SPME) technique with on-fiber derivatization for measuring carbonyl emissions from e-cigarettes. A 2-cm triphasic divinylbenzene/carboxen/polydimethylsiloxane fiber was used and derivatized carbonyls were measured by GC-MS. The puff volume was 70 mL volume, the puff duration varied from 2 to 10 s and the interpuff interval was 20 s. Two types of second-generation e-cigarettes were tested and carbonyl emissions were reported as amount per mL liquid consumption. Differences were observed between devices, with formaldehyde reaching up to 135 μg/mL, acetaldehyde up to 170 μg/mL and acrolein up to 1.3 μg/mL (approximate values derived from figures that did not report the exact values). The authors also reported that puff duration positively corrected with acetaldehyde and acrolein emissions.

Klager et al. (2017) analyzed the aerosol of 26 first generation e-cigarettes for carbonyl emissions. The puffing regime was 45–80 mL volume (volume levels necessary for the automatic activation of the devices), 2 s duration, and 60 s interpuff interval. No puff number was mentioned, but the authors reported that the aerosol was sampled for ~3 h. Silica sorbent tubes were used for aerosol collection and the analysis was performed with HPLC-UV. Levels were reported in μg/m^3^, with formaldehyde ranging from below LOQ to 10,900 μg/m^3^, acetaldehyde from 22.5 to 20,400 μg/m^3^, and crotonaldehyde from below LOQ to 82,900 μg/m^3^. Unlike the findings by Khlystov and Samburova (2016), no correlation between flavoring compounds and carbonyl emissions was observed in this study.

Farsalinos K. E. et al. (2017b) performed a replication of the study by Jensen et al. (2015) using the same e-cigarette battery device, atomizer and liquid. The authors recruited experienced vapers to identify the voltage setting associated with overheating (dry puffs) and then tested the device at different voltage settings under both realistic (3.3, 3.6, 4.0 V) and dry puff conditions (4.2, 4.6, 4.8, and 5.0 V). The puffing regime was 60 mL volume, 4 s duration, and 30 s interpuff interval. Aerosol was collected in two impingers containing DNPH that were connected in series and analysis was performed using HPLC-UV. Formaldehyde levels ranged from 3.4 μg/10 puffs at 3.3 V to 718.2 μg/10 puffs at 5.0 V. Compared to the findings by Jensen et al. (2015), formaldehyde levels were detected at 3.3 V and were 89% higher at 5.0 V, verifying that high formaldehyde emissions previously reported. At the upper limit of dry puff conditions, formaldehyde levels were 19.8 μg/10 puffs (1005.4 μg/3 g liquid consumption), a level 36-fold lower compared to 5.0 V. The authors concluded that very high formaldehyde levels emitted at high voltage settings are associated with dry puffs and thus are not relevant to true human exposure. The authors also noted that the atomizer used was an outdated and inefficient design that is no longer available in the European Union.

Beauval et al. (2017) tested a second generation e-cigarette device with 6 liquids (2 flavored and 1 unflavored, with and without nicotine) for carbonyl emissions. The puffing regime was 55 mL volume, 3 s duration and 30 s interpuff interval. Aerosol passed through silica cartridges coated with DNPH and analysis was performed with HPLC-DAD. Carbonyl levels were expressed as amount per mL puff volume, with formaldehyde ranging from 0.37 to 1.48 ng/mL, acetaldehyde from 0.16 to 0.96 ng/mL and acrolein from non-detected to 2.11 ng/mL.

Talih et al. (2017) evaluated 2 “sub-ohm” atomizers (low resistance value of the coil), which are normally used in a “direct lung inhalation” pattern of e-cigarette use (users inhale directly from the e-cigarette into the lung instead of keeping the aerosol in the oral cavity during puff intake and subsequently inhaling it). They used high power (50, 75, and 100 W), which is necessary to generate aerosol with these devices. Another conventional (“mouth to lung”) device tested at 4 and 11 W was used for comparison. The puffing regime was 66.7 mL volume, 4 s duration and 10 s interpuff interval. Aerosol passed through DNPH-coated silica cartridges and analysis was performed by HPLC-UV. Formaldehyde ranged from 5.1 to 24.19 μg/15 puffs (0.34 to 1.62 μg/puff), acetaldehyde from 8.36 to 25.06 μg/15 puffs (0.56 to 1.67 μg/puff), acetone from 2.34 to 55.41 μg/15 puffs (0.16 to 3.68 μg/puff) and acrolein from non-detected to 1.34 μg/15 puffs (0.09 μg/puff).

Farsalinos K. E. et al. (2017a) performed another replication, testing the same e-cigarette device and liquid at the same puffing patterns and voltage settings (3.8 and 4.8 V) as Sleiman et al. (2016). Additionally, they tested another, newer-generation, atomizer at two power settings (9 and 13.5 W) and different puffing regime which, according to the authors, represented a more realistic pattern. Two experienced vapers tested the devices to identify whether the testing conditions were associated with overheating (dry puffs). The puffing regime for the replication part of the study was 50 mL volume, 5 s duration, and 30 s interpuff interval. For the newer generation atomizer, the puffing regime was 50 mL volume, 4 s duration, and 30 s interpuff interval. Aerosol was collected in one impinger containing DNPH and analysis was performed using HPLC-UV. Dry puffs were identified in the replication experiment at both voltage settings. Formaldehyde levels ranged from 796.7 to 4259.6 μg/ g liquid, acetaldehyde from 320.6 to 2156.2 μg/ g liquid and acrolein from 69.1 to 623.6 μg/g liquid at 3.8 and 4.8 V, respectively. Compared to the findings by Sleiman et al. (2016), formaldehyde levels were detected at ~11-fold lower levels, acetaldehyde at 6- to 9-fold lower levels and acrolein at 16- to 25-fold lower levels. The newer generation atomizer did not generate dry puffs and emitted formaldehyde at 16.7 and 16.5 μg/g liquid, acetaldehyde at 9.6 and 10.3 μg/g liquid and acrolein at 8.6 and 11.7 μg/g liquid at 9 and 13.5 W, respectively. These levels represented a 94.4–99.8% lower carbonyl exposure from consuming 5 g of liquid compared to smoking 20 cigarettes per day. Of note, no statistically significant difference in carbonyl emissions was observed between low and high power settings. The authors explained that this was due to reporting the levels per amount of liquid consumption, showing that the thermal degradation rate of the liquid did not increase at high power settings. The authors also reported that carbonyl emissions from the newer generation atomizer were lower than commonly measured environmental levels (indoor air) and occupational safety limits.

Kosmider et al. (2017) analyzed carbonyl emissions from a newer generation atomizer and a liquid at two nicotine concentrations (6 and 24 mg/mL) using puffing patterns that were recorded in experienced vapers previously (Dawkins et al., 2016). Carbonyls were trapped in tubes packed with solid adsorbent and analysis was performed by HPLC with diode array detector (HPLC-DAD) and levels were reported as amount per puff and amount per 1 h consumption (based on the puffing topography recordings in vapers). Levels of carbonyls were lower when using the 24 mg/mL compared to the 6 mg/mL nicotine concentration liquid, with formaldehyde levels ranging from 1.49 to 3.41 μg/h, acetaldehyde from 1.59 to 3.31 μg/h and acetone from 0.28 to 0.73 μg/h, respectively. Acrolein was not detected in any samples. The authors reported that the levels of aerosol yield per puff based on the puffing patterns recorded in vapers were 11.1 mg for the 6 mg/mL and 7.3 mg for the 24 mg/mL nicotine concentration liquid.

## Discussion—methodological considerations

The issue of carbonyl emissions from e-cigarettes has generated a lot of research interest. This is understandable both because carbonyls are important toxicants and because it is reasonable to expect carbonyls to be formed and emitted through the thermal degradation of e-cigarette liquid ingredients. This systematic review identified several discrepancies in research conducted until now and raises several methodological considerations that need to be addressed to improve the quality and usefulness of future research.

A major characteristic observed from this review is the diversity of puffing regimes, carbonyl trapping materials, analytical methods, and reported units of measurements (Tables 1, 2). This is expected due to the lack of standardized puffing patterns. Of particular importance, 22 distinct puffing regimes were identified. Puff volume ranged from 33.4 to 152.8 mL, with most studies using volumes from 40 to 70 mL. Puff volume is not expected to affect carbonyl emissions when within a reasonable range. However, it should be noted that one study (Talih et al., 2017) used inappropriately low puff volume (66.7 mL) for atomizers that are used for direct lung inhalation. Direct lung inhalation is associated with puff volumes by far exceeding tidal volume, with anecdotal measurements (performed by the authors of this review) up to 1.5 L per puff or more. Such difference could affect the temperature in the coil and, thus, the thermal degradation rate of liquid ingredients, leading to findings which are not applicable to true human exposure. Puff duration ranged from 1.8 to 8 s, with most studies using duration from 2 to 4 s. Puff duration is an important parameter in temperature generation since it directly affects the energy delivery to the atomizer. Although puffing topography studies have identified a range from 2 to 4 s as a reasonable choice (Farsalinos et al., 2013a; Hua et al., 2013), it should be noted that this parameter is quite complex. Nicotine concentration in liquids and power setting of devices are known factors that affect puff duration (Dawkins et al., 2016; Lopez et al., 2016; Farsalinos K. et al., 2017a). The latter is relevant to the newer generation e-cigarette products, the vast majority of which are variable power devices. Nicotine delivery to the aerosol is also dependent on atomizer performance characteristics and varies between atomizers even when using the same liquid (Farsalinos et al., 2016). Thus, it is likely that a standardized puff duration is not appropriate for testing all available e-cigarette products; for example, it has been proposed that an approach of reducing puff duration at high power in laboratory studies would be more relevant to realistic human use (Farsalinos K. et al., 2017b). Interpuff interval ranged from 10 to 60 s, with most studies using 30 s. The latter is probably a reasonable choice. The 10 s interpuff interval was chosen based on observations in users (Goniewicz et al., 2014), however they probably used first generation devices with limited power and performance and they were also taking short puffs (1.8 s). In one study, 10 s interpuff interval was used while obtaining 8 s puffs (Talih et al., 2016), both of which represent extreme patterns and probably not representative of average use. The interpuff interval may affect the temperature of evaporation since e-cigarettes generate heat only when activated while on puff termination the temperature gradually decreases toward environmental levels. A short interpuff interval may result in higher baseline temperature at the time of the next puff initiation, and this could affect the maximum temperature and the overall thermal load. A potential result of a very short interpuff interval could be the generation of dry puffs, discussed below.

**Table 1:** Puffing regimes, carbonyl trapping materials, analytical methods, and units reported in studies (*n* = 32) measuring carbonyl emissions from e-cigarettes.

**Characteristic**	**Number of studies**	**Studies**
**PUFFING REGIME^a^**		
55/2/30	3	Uchiyama et al., 2013, 2016; Tayyarah and Long, 2014
70/1.8/10	1	Goniewicz et al., 2014
70/1.8/17	1	Kosmider et al., 2014
55/3/30	2	Hutzler et al., 2014; Beauval et al., 2017
35/4/30	1	Geiss et al., 2015
50/4/30	2	Jensen et al., 2015; Farsalinos K. E. et al., 2017a
70/3/10	1	Laugesen, 2015
60/4/30	2	Farsalinos et al., 2015; Farsalinos K. E. et al., 2017b
40/4/10	1	Herrington and Myers, 2015
43/2/15-60	1	Blair et al., 2015
152.8/8/10	1	Talih et al., 2016
55/4/30	3	Flora et al., 2016, 2017; Gillman et al., 2016
33.4/2/10	1	Jo and Kim, 2016
50/3/20	1	Geiss et al., 2016
80/4/30	1	Havel et al., 2017
50/5/30	2	Sleiman et al., 2016; Farsalinos K. E. et al., 2017a
100/4/10	1	El-Hellani et al., 2016
40/4/30	1	Khlystov and Samburova, 2016
91/4/30	1	Ogunwale et al., 2017
70/2/10	1	Sala et al., 2017
45-80/2/60	1	Klager et al., 2017
66.7/4/10	1	Talih et al., 2017
**CARBONYL TRAPPING MATERIALS**		
DNPH-coated silica cartridges/silica sorbent tubes	13	Goniewicz et al., 2014; Kosmider et al., 2014; El-Hellani et al., 2016; Geiss et al., 2016; Jo and Kim, 2016; Khlystov and Samburova, 2016; Sleiman et al., 2016; Talih et al., 2016, 2017; Beauval et al., 2017; Klager et al., 2017; Kosmider et al., 2017; Wang et al., 2017
Hydroquinone-DNPH coupled silica cartridges	2	Uchiyama et al., 2010, 2013
Impingers with DNPH	10	Hutzler et al., 2014; Tayyarah and Long, 2014; Farsalinos et al., 2015; Farsalinos K. E. et al., 2017a,b; Laugesen, 2015; Flora et al., 2016, 2017; Gillman et al., 2016; Havel et al., 2017
Tedlar bags and DNPH-coated silica cartridges	1	Geiss et al., 2015
NMR spectroscopy tube	1	Jensen et al., 2015
Thermal desorption tubes	1	Herrington and Myers, 2015
Teflon bag and fast flow tube	1	Blair et al., 2015
Sorbent cartridge with Carboxen-572 particles	1	Uchiyama et al., 2016
Tedlar bag and silicon microreactors with AMAH	1	Ogunwale et al., 2017
Divinylbenzene/carboxen/polydimethylsiloxane fiber	1	Sala et al., 2017
**ANALYTICAL METHOD**		
HPLC	24	Uchiyama et al., 2010, 2013, 2016; Goniewicz et al., 2014; Hutzler et al., 2014; Kosmider et al., 2014; Farsalinos et al., 2015; Farsalinos K. E. et al., 2017a,b; Geiss et al., 2015, 2016; Laugesen, 2015; El-Hellani et al., 2016; Gillman et al., 2016; Jo and Kim, 2016; Khlystov and Samburova, 2016; Sleiman et al., 2016; Talih et al., 2016, 2017; Beauval et al., 2017; Havel et al., 2017; Klager et al., 2017; Kosmider et al., 2017; Wang et al., 2017
UPLC	3	Tayyarah and Long, 2014; Flora et al., 2016, 2017
NMR spectroscopy	1	Jensen et al., 2015
TD-GC-MS	1	Herrington and Myers, 2015
PTRMS	1	Blair et al., 2015
GC-MS, NMR	1	Ogunwale et al., 2017
SPME-GC-MS	1	Sala et al., 2017
**REPORTED UNITS^b^**		
Amount per aerosol volume (m^3^ or L or mL)	5	Uchiyama et al., 2010, 2013; Laugesen, 2015; Beauval et al., 2017; Klager et al., 2017
Amount per puff number	20	Goniewicz et al., 2014; Hutzler et al., 2014; Kosmider et al., 2014; Tayyarah and Long, 2014; Blair et al., 2015; Farsalinos et al., 2015; Farsalinos K. E. et al., 2017b Geiss et al., 2015, 2016; Jensen et al., 2015; El-Hellani et al., 2016; Flora et al., 2016, 2017; Gillman et al., 2016; Khlystov and Samburova, 2016; Talih et al., 2016, 2017; Uchiyama et al., 2016; Havel et al., 2017; Ogunwale et al., 2017
Amount per liquid consumption	8	Gillman et al., 2016; Jo and Kim, 2016; Khlystov and Samburova, 2016; Sleiman et al., 2016; Sala et al., 2017; Wang et al., 2017; Farsalinos K. E. et al., 2017a,b
Ppm	1	Herrington and Myers, 2015

a *One study (Wang et al., 2017) did not use an e-cigarette to generate aerosol, and another study (Uchiyama et al., 2010) did not report puff duration and interpuff interval. Thus, puffing regime is not identified in these studies. One study (Farsalinos K. E. et al., 2017a) tested two e-cigarette atomizers at different puffing regimes. One study (Kosmider et al., 2017) tested puffing regimes based on topography recordings in experienced vapers; the puffing regimes are not displayed in the table*.

b *Some studies reported more than one unit for carbonyl emissions. One study (Kosmider et al., 2017) reported aerosol emissions as amount per hour of e-cigarette use*.

**Table 2:** Puff volume, puff duration, and interpuff interval used in studies (*n* = 32) measuring carbonyl emissions from e-cigarettes.

**Puffing parameter**	**Number of studies^a^**	**Studies**
**PUFF VOLUME**		
33.4 mL	1	Jo and Kim, 2016
35 mL	1	Geiss et al., 2015
40 mL	2	Herrington and Myers, 2015; Khlystov and Samburova, 2016
43 mL	1	Blair et al., 2015
50 mL	4	Jensen et al., 2015; Geiss et al., 2016; Sleiman et al., 2016; Farsalinos K. E. et al., 2017a
55 mL	8	Uchiyama et al., 2013, 2016; Hutzler et al., 2014; Tayyarah and Long, 2014; Flora et al., 2016, 2017; Gillman et al., 2016; Beauval et al., 2017
60 mL	2	Farsalinos et al., 2015; Farsalinos K. E. et al., 2017b
66.7 mL	1	Talih et al., 2017
70 mL	4	Goniewicz et al., 2014; Kosmider et al., 2014; Laugesen, 2015; Sala et al., 2017
80 mL	1	Havel et al., 2017
91 mL	1	Ogunwale et al., 2017
100 mL	1	El-Hellani et al., 2016
152.8 mL	1	Talih et al., 2016
Variable	1	Klager et al., 2017
**PUFF DURATION**		
1.8 s	2	Goniewicz et al., 2014; Kosmider et al., 2014
2 s	6	Uchiyama et al., 2013, 2016; Tayyarah and Long, 2014; Blair et al., 2015; Jo and Kim, 2016; Klager et al., 2017
3 s	4	Hutzler et al., 2014; Laugesen, 2015; Geiss et al., 2016; Beauval et al., 2017
4 s	14	Havel et al., 2017; Farsalinos et al., 2015; Farsalinos K. E. et al., 2017a,b Geiss et al., 2015; Herrington and Myers, 2015; Jensen et al., 2015; El-Hellani et al., 2016; Flora et al., 2016, 2017; Gillman et al., 2016; Khlystov and Samburova, 2016; Ogunwale et al., 2017; Talih et al., 2017
5 s	2	Sleiman et al., 2016; Farsalinos K. E. et al., 2017a
8 s	1	Talih et al., 2016
**INTERPUFF INTERVAL**		
10 s	7	Goniewicz et al., 2014; Herrington and Myers, 2015; Laugesen, 2015; El-Hellani et al., 2016; Jo and Kim, 2016; Talih et al., 2016, 2017
17 s	1	Kosmider et al., 2014
20 s	1	Geiss et al., 2016
30 s	17	Uchiyama et al., 2013, 2016; Hutzler et al., 2014; Tayyarah and Long, 2014; Farsalinos et al., 2015; Farsalinos K. E. et al., 2017a,b Geiss et al., 2015; Jensen et al., 2015; Flora et al., 2016, 2017; Gillman et al., 2016; Khlystov and Samburova, 2016; Sleiman et al., 2016; Beauval et al., 2017; Havel et al., 2017; Ogunwale et al., 2017
60 s	2	Blair et al., 2015; Klager et al., 2017

a *One study (Wang et al., 2017) did not use an e-cigarette to generate aerosol, and another study (Uchiyama et al., 2010) did not report puff duration and interpuff interval. Thus, puffing regime is not identified in these studies. One study (Kosmider et al., 2017) tested puffing regimes based on topography recordings in experienced vapers; the puffing regimes are not displayed in the table. One study (Sala et al., 2017) used variable puff duration, ranging from 2 to 10 s. One study (Blair et al., 2015) used variable interpuff interval, ranging from 15 to 60 s*.

Another issue relevant to the choice of puff duration and the power settings used in the laboratory setting is the dry puff phenomenon. This is an organoleptic (sensory) parameter of unpleasant (“burning”) taste related to overheating of liquids that is widely known and reported by e-cigarette users. It was first mentioned in the scientific literature in 2013 (Farsalinos et al., 2013a; Romagna et al., 2013), and was presented in detail in 2015 (Farsalinos et al., 2015). Overheating happens when there is an imbalance between liquid supply to the wick of the atomizer head and energy delivery to the coil. Energy delivered from the battery device is transformed to heat needed to increase the temperature of the liquid so that it evaporates. The system eventually reaches to a balance where a specific temperature is maintained and liquid evaporates throughout the puff (Soulet et al., 2017). When there is not enough liquid on the coil to maintain that balance, more energy is transformed to heat further increasing the temperature of the coil and increasing the thermal degradation rate of liquid ingredients. Conditions such as low levels of liquid in the atomizer, too much energy delivered relevant to the atomizer head design (too much power and/or puff duration), or limited liquid supply to the coil (e.g., due to liquids with high viscosity) can create an imbalance. Atomizer design features such as mass and surface area of the heating coil, volume and material of the wick and liquid feeding system to the wick determine the ideal energy (power × duration) range for each atomizer, which obviously varies between different products. The ability of e-cigarette batteries to deliver a large range of power does not mean that all atomizers can be used at any power setting. Since dry puffs are detected and avoided by e-cigarette users due to the unpleasant taste and experience, it is important for laboratory studies to ensure that dry puffs are not generated during aerosol generation for emission testing. Since this is a subjective sensory parameter, only experienced user can determine generation of dry puffs, when testing the e-cigarette at the same conditions (puff duration, interpuff interval, and power settings) as tested in the laboratory. Dry puffs are more likely to occur when variable power e-cigarette battery devices are tested. Unfortunately a very small number of studies ensured that no dry puffs were generated under the conditions tested or tested for the generation of dry puffs by recruiting e-cigarette users (Farsalinos et al., 2015; Farsalinos K. E. et al., 2017a,b; Geiss et al., 2016). It should be noted that the studies performed under verified realistic use conditions showed that carbonyl emissions from e-cigarettes were by far lower than tobacco cigarette smoke. There are indications from several studies that dry puff conditions were generated during aerosol testing. Hutzler et al. (2014) used the e-cigarette device until no visible aerosol was emitted, which is a condition clearly associated with dry puffs. It has already been documented that the findings by Jensen et al. (2015) that e-cigarettes emit 5–15 times higher formaldehyde levels compared to smoking were related to extreme dry puff conditions (Farsalinos K. E. et al., 2017b). Sleiman et al. (2016) found unusually high carbonyl emissions from e-cigarettes (up to 48 mg/g formaldehyde and 19 mg/g acetaldehyde), which also raised the possibility of dry puffs. Of note, the formaldehyde levels detected correspond to exposure from using 5 g liquid (an average daily consumption for e-cigarette users) being equivalent to smoking >3,500 tobacco cigarettes (Counts et al., 2005). The authors subsequently performed a risk assessment analysis and identified, as expected, high levels of exposure and risk to consumers (Logue et al., 2017). This study was replicated by Farsalinos K. E. et al. (2017a) and identified both the generation of dry puffs and a substantial overestimation of carbonyl emissions. Therefore, the study measuring carbonyl emissions and the subsequent risk assessment analysis have no clinical relevance. In fact, carbonyl emissions can be produced “on demand,” simply by overheating the devices to extreme temperatures. The temperature can reach to levels approximating 1,000°C when no liquid is present in the wick (Geiss et al., 2016), and it is expected that carbonyl emissions will increase by orders of magnitude at these temperatures. Therefore, ensuring that dry puffs are avoided is essential when examining carbonyl (and other thermal degradation) emissions in the context of realistic human exposure.

There have been several different analytical approaches for the measurement of aldehydes in e-cigarette aerosol but the most common method includes the use of DNPH to produce stable and easily measureable DNPH-adducts. DNPH based methods have been widely used to the analysis of tobacco smoke (CORESTA, 2014) and have been shown to be fit for purpose for a wide range of sample matrixes (USEPA, 1999). However, DNPH based methods do have potential limitations. Coated sorbent tubes have been shown to have poor performance for the measurement of unsaturated aldehydes like acrolein (Ho et al., 2011). Additionally, one study tested several DNPH-coated cartridges with e-cigarettes and found that some created significant pressure drop (Geiss et al., 2016), which could impede the airflow through the atomizer and result in overheating that a user would not experience under realistic use. Importantly, DNPH reacts readily with a wide range of aldehydes and ketones, not just formaldehyde, acetaldehyde, and acrolein which may lead to reporting of inaccurate results. Considering that e-cigarette aerosols are complex mixtures with flavorings containing several compounds, including non-toxic aldehydes, there is the possibility for false-positive results and misidentification of aldehyde flavoring compounds as toxic carbonyls. The range compounds that might be present in a particular flavored e-liquid makes it very difficult to accurately determine carbonyl compounds produced by the thermal decomposition of PG and VG. Analytical methods for use with e-cigarettes are typically validated using just a few, if any, flavored e-liquids, and since is not possible for method validations to include the full range of commercially available products, researchers are cautioned to confirm atypical results using at least one alternate analytical method. Alternate analytical methods also have drawbacks. Since -cigarette aerosols are a complex mixture of semi-liquid particles (Ingebrethsen et al., 2012), collection in Tedlar bags may lead to sample loss due to condensation. Other analysis methods including GC-MS and NMR are not widely used and method validation details have not been published for these new methods. Results for new or novel methods should always be compared with established methodologies.

The levels of carbonyl emissions are typically reported as amount per puff number. Although this could be relevant to tobacco cigarette research, such reporting in e-cigarettes has a major limitation when comparing different power settings or puff durations. It does not take into account that aerosol yield (liquid consumption) per puff increases substantially at higher power settings (Gillman et al., 2016) or with higher puff durations (Talih et al., 2015). Even if the thermal degradation rate (percent of liquid that is transformed to aldehydes) remains stable, the higher liquid consumption per puff will inevitably increase the absolute levels of carbonyls per puff, but not necessarily the amount per liquid consumption. Since surveys of vapers have shown that electronic cigarette use consumption is measured as liquid consumption per day rather than number of puffs (Dawkins et al., 2013; Farsalinos et al., 2013b, 2014), reporting the level of emissions per liquid consumption rather than puffs is essential and relevant to true exposure. In fact, all e-cigarette aerosol emissions should ideally be reported as amount per liquid consumption, and liquid consumption is probably the main determinant of emissions exposure. Characteristically, Kosmider et al. (2017) reported higher carbonyl exposure when using 6 mg/mL compared to 24 mg/mL liquid, based on puffing patterns and liquid consumption during a 1 h session in experienced vapers. However, by calculating the levels of aldehyde emissions per gram of liquid, based on the information on aerosol yield per puff, slightly higher formaldehyde (4.343 μg/g vs. 4.153 μg/g) and acetaldehyde (3.027 μg/g vs. 2.640 μg/g) were observed at 24 mg/mL compared to 6 mg/mL nicotine concentration liquid. This clearly shows that it is the higher liquid consumption at 6 mg/mL that mainly determines the higher carbonyl exposure in users. Reporting carbonyl emissions as mg/m^3^ could be relevant to environmental emissions (second-hand exposure) but is problematic when assessing exposure to users due to the intermittent nature of e-cigarette use.

Some studies produced contradictory results. Kosmider et al. (2014) found that VG liquids emitted lower carbonyl emissions compared to PG liquids. Geiss et al. (2015) found that VG liquids remitted higher carbonyl levels compared to mixed PG/VG liquids and Wang et al. (2017) found higher levels of carbonyls in VG compared to PG liquids. VG has higher viscosity compared to PG and, unless diluted with water, it is possible that this might adversely affect the liquid supply rate to the coil and, thus, create overheating conditions. This is an issue that needs to be further studied. Discrepancies were observed in the temperature associated with marked elevation of carbonyl emissions. Hutzler et al. (2014) found a steep elevation of carbonyl emissions at 150°C, Wang et al. (2017) at 270°C, Geiss et al. at >300°C and Flora et al. 350°C. Of all these studies, only Geiss et al. (2016) and Flora et al. (2016) measured temperature in an e-cigarette, and it is possible that the temperatures in these studies were associated with dry puffs. It is currently unclear if under realistic use conditions there is a critical temperature point above which carbonyl emissions increase substantially.

One study that deserves specific mention found that flavoring compounds are the main source of carbonyl emissions from e-cigarettes (Khlystov and Samburova, 2016). In some flavored liquids, very high levels of carbonyls were detected (up to ~7 mg/g formaldehyde and 3.5 mg/g acetaldehyde). The authors did not detect carbonyl emissions in unflavored liquids, while up to 10,000-fold higher emissions were detected in flavored liquids (Farsalinos K. et al., 2017a). A letter to the editor commented that other studies which evaluated flavored and unflavored liquids failed to detect such large differences in carbonyl emissions Farsalinos K. et al. (2017a). Klager et al. (2017) found no correlation between flavoring compounds and carbonyl emissions. Since most e-cigarette users use flavored liquids, a finding that flavorings are the main source of carbonyls and result in substantial carbonyl emissions (e.g., more than 7 mg/g formaldehyde) has significant public health implications. Thus, it is extremely important for the study to be replicated and research should expand on different flavorings in an attempt to identify potential compounds that could contribute to high carbonyl emissions.

Finally, it should be mentioned that three studies which assessed newer generation atomizers (tank systems using cotton wick) found that carbonyl emissions were extremely low even at high power settings (Gillman et al., 2016; Farsalinos K. E. et al., 2017a; Kosmider et al., 2017). An important characteristic of these devices is that the atomizer head is located at the bottom of the tank (“bottom coil”) thus facilitating the liquid replenishment due to the effects of gravity. Additionally, they contain cotton, instead of silica, wick, which has more sorptivity and is more porous thus further enhancing the liquid supply to the heat source. Gillman et al. (2016) also tested old generation atomizers and found substantially higher levels of carbonyl emissions. This study indicated that the development of new atomizers with better wicking material results in improvement of not only the performance characteristics (more aerosol yield per puff) but also the safety profile of the devices. In fact, carbonyl emissions from the newer generation atomizers were not just lower than tobacco cigarettes but lower than commonly measured environmental levels and occupational safety limits. For example, the World Health Organization (2010) reports that indoor air of homes can have up to 250 μg/m^3^ formaldehyde, although on average levels of <50 μg/m^3^ are found. Considering a daily ventilation volume of 20 m^3^/d, the daily formaldehyde exposure from breathing indoor air is ~1,000 μg, by far higher than the total exposure from consuming 5 g of the liquid using the newer generation atomizer tested by Farsalinos K. E. et al. (2017a) which was found to be 83.3 μg. Such levels of emissions are of questionable clinical significance in terms of health risk. It should be mentioned, however, that the overall risk related to e-cigarette use is not solely linked to carbonyl emissions but to the emission of other compounds that could have a toxicological potential. Further studies should specifically examine how new wicking materials affect the evaporation process, temperature of evaporation and thermal degradation of liquid ingredients.

## Conclusion

Carbonyl emissions in e-cigarettes represent an important research topic that has generated a lot of interest. The present review identified different methodologies used in the laboratory assessment of carbonyl emissions. Of particular concern is the large diversity of puffing patterns used, which makes comparisons difficult while in some cases the puffing regime was unrealistic. While varying puffing patterns is understandable considering the diversity of e-cigarette device performance and functional characteristics, it seems that choice of puffing regimes was not based on these parameters. The variability of reported units of carbonyl emissions can also create confusion and may be difficult to interpret. A reasonable recommendation would be to report values per amount of liquid consumption. Additionally, analytical methods need to be accurately validated since the possibility of false positive and false negative results is of concern due to the complexity of ingredients in flavored liquids. Finally, it is particularly important that laboratory studies ensure that no dry puffs are generated under laboratory conditions; otherwise testing realistic conditions relevant to true human exposure cannot be ensured and the findings could be misleading and misinformative for consumers and regulators. A result of these research discrepancies is that the reported carbonyl emissions varied from extremely low (lower not only compared to tobacco cigarette but also compared to environmental levels) to extremely high (up to orders of magnitude higher than tobacco cigarettes. Further research should consider all these concerns in order to improve research quality and find ways to reduce thermal degradation and carbonyl emissions from e-cigarettes.

## Author contributions

All authors listed have made a substantial, direct, and intellectual contribution to the work, and approved it for publication.

### Conflict of interest statement

In the past 3 years, KF has published 2 studies funded by the non-profit association AEMSA and 1 study funded by the non-profit association Tennessee Smoke-Free Association. Enthalpy Analytical is a for-profit CRO involved in analytical testing of tobacco and e-cigarette products. The other author declares that the research was conducted in the absence of any commercial or financial relationships that could be construed as a potential conflict of interest.
